# Antimicrobial Photodynamic Therapy with Chlorin e6 Is Bactericidal against Biofilms of the Primary Human Otopathogens

**DOI:** 10.1128/mSphere.00492-20

**Published:** 2020-07-15

**Authors:** Nicole R. Luke-Marshall, Lisa A. Hansen, Gal Shafirstein, Anthony A. Campagnari

**Affiliations:** a Department of Microbiology and Immunology, University at Buffalo, Buffalo, New York, USA; b Photodynamic Therapy Center, Roswell Park Comprehensive Cancer Center, Buffalo, New York, USA; Antimicrobial Development Specialists, LLC

**Keywords:** antimicrobial photodynamic therapy, biofilm, chlorin e6, otitis media, otopathogens

## Abstract

Moraxella catarrhalis, Streptococcus pneumoniae, and nontypeable Haemophilus influenzae (NTHi) are ubiquitous upper respiratory opportunistic pathogens. Together, these three microbes are the most common causative bacterial agents of pediatric otitis media (OM) and have therefore been characterized as the primary human otopathogens. OM is the most prevalent bacterial infection in children and the primary reason for antibiotic administration in this population. Moreover, biofilm formation has been confirmed as a primary mechanism of chronic and recurrent OM disease. As bacterial biofilms are inherently metabolically recalcitrant to most antibiotics and these complex structures also present a significant challenge to the immune system, there is a clear need to identify novel antimicrobial approaches to treat OM infections. In this study, we evaluated the potential efficacy of antibacterial photodynamic therapy (aPDT) with the photosensitizer chlorin e6 (Ce6) against planktonic as well as biofilm-associated M. catarrhalis, S. pneumoniae, and NTHi. Our data indicate aPDT with Ce6 elicits significant bactericidal activity against both planktonic cultures and established biofilms formed by the three major otopathogens (with an efficacy of ≥99.9% loss of viability). Notably, the implementation of a novel, dual-treatment aPDT protocol resulted in this disinfectant effect on biofilm-associated bacteria and, importantly, inhibited bacterial regrowth 24 h posttreatment. Taken together, these data suggest this novel Ce6-aPDT treatment may be a powerful and innovative therapeutic strategy to effectively treat and eradicate bacterial OM infections and, significantly, prevent the development of recurrent disease.

**IMPORTANCE** Otitis media (OM), or middle ear disease, is the most prevalent bacterial infection in children and the primary reason for antibiotic use and surgical intervention in the pediatric population. Biofilm formation by the major bacterial otopathogens, Moraxella catarrhalis, Streptococcus pneumoniae, and nontypeable Haemophilus influenzae, has been shown to occur within the middle ears of OM patients and is a key factor in the development of recurrent disease, which may result in hearing impairment and developmental delays. Bacterial biofilms are inherently impervious to most antibiotics and present a significant challenge to the immune system. In this study, we demonstrate that antimicrobial photodynamic therapy (aPDT) using the photosensitizer chlorin e6 elicits significant bactericidal activity versus planktonic and biofilm-associated otopathogens and supports further analyses of this novel, efficacious, and promising technology as an adjunctive treatment for acute and recurrent OM.

## Abstract (precis)

Otitis media (OM), or middle ear disease, is the most prevalent bacterial infection in children and the primary reason for antibiotic use and surgical intervention in the pediatric population. Biofilm formation by the major bacterial otopathogens, Moraxella catarrhalis, Streptococcus pneumoniae, and nontypeable Haemophilus influenzae, has been shown to occur within the middle ears of OM patients and is a key factor in the development of recurrent disease, which may result in hearing impairment and developmental delays. Bacterial biofilms are inherently impervious to most antibiotics and present a significant challenge to the immune system. In this study, we demonstrate that antimicrobial photodynamic therapy (aPDT) using the photosensitizer chlorin e6 elicits significant bactericidal activity versus planktonic and biofilm-associated otopathogens and supports further analyses of this novel, efficacious, and promising technology as an adjunctive treatment for acute and recurrent OM.

## INTRODUCTION

Otitis media (OM), or middle ear disease, is the most prevalent pediatric bacterial infection as well as the single most common reason for antibiotic use in young children. Not only has this treatment approach resulted in a rapid increase in microbial resistance, but recent studies have suggested that frequent antimicrobial use early in life, often due to recurrent OM, is correlated with the subsequent development of allergic diseases during childhood (1, 2). In addition, repeated episodes of OM are associated with the presence of bacterial biofilms. Biofilm formation by mucosal pathogens is a critical factor in the establishment of persistent colonization and the subsequent manifestations of chronic disease, and this correlation has been well documented in OM (3). Three predominate human upper respiratory tract bacterial commensals, Streptococcus pneumoniae, nontypeable Haemophilus influenzae (NTHi), and Moraxella catarrhalis, account for the majority of both acute and chronic bacterial OM episodes (recently reviewed in references 4 and 5). Moreover, biofilms composed of these otopathogens have been identified in clinical samples obtained from adenoid and middle ear tissues of pediatric OM patients (6–8).

Bacterial biofilms are inherently metabolically recalcitrant to standard antibiotic regimens, and these complex structures also present a significant challenge to the host immune system. There is clearly a critical need for the identification of highly efficacious alternative antimicrobial approaches to treat and eliminate chronic/recurrent biofilm-based infections such as OM. Optimally, these new treatment modalities should employ killing mechanisms that do not facilitate the development of microbial resistance. A promising novel strategy for targeting biofilm-associated infections, including OM, is antimicrobial photodynamic therapy (aPDT) (recently reviewed in reference 9). aPDT uses photosensitizers (PSs) to generate cytotoxic reactive oxygen species (ROS) upon irradiation by light at PS-specific wavelengths. Localization of the PS to the biofilm matrix, bound to the prokaryotic cell surface, or within the cytoplasm results in nonspecific multitarget oxidative damage of adjacent biomolecules (e.g., nucleic acids, proteins, lipids, and polysaccharides) upon illumination. The resulting production of ROS induces nonrepairable and lethal oxidative damage affecting the integrity of the cell surface and intracellular biomolecules essential for viability as well as significant destruction and disintegration of the biofilm matrix (9). In contrast to antibiotics, aPDT represents a particularly attractive alternative antimicrobial strategy to combat and eradicate OM infections as it causes nonspecific bactericidal effects, which can be confined to a defined anatomical location based on localized PS and light delivery. We previously reported aPDT using the PS porfimer sodium elicits significant bactericidal activity versus planktonic as well as biofilm-associated M. catarrhalis (10). However, an aPDT protocol that exhibits significant bactericidal activity against all three major otopathogens was not evaluated.

Thus, the objective of the present study was to elucidate the antibacterial efficacy of aPDT using the PS chlorin e6 (Ce6) and blue light (405 nm) against biofilm-grown and planktonic cells of the three major bacterial otopathogens, M. catarrhalis, NTHi, and S. pneumoniae. Unlike porfimer sodium, which has therapeutic limitations such as prolonged patient phototoxicity, the amphiphilic chlorophyll derivative Ce6 has been shown to be a promising candidate for systemic and localized treatment modalities, as it exhibits highly effective PDT activities (anticancer and antimicrobial) in the absence of significant dark toxicity (11, 12). The data presented in this report demonstrate Ce6-aPDT elicits significant bactericidal activity versus the three primary bacterial otopathogens, suggesting that this technology warrants further analysis as a novel antimicrobial treatment for the prevention of recurrent OM.

## RESULTS

### aPDT of otopathogens with Ce6.

**FIG 1:**
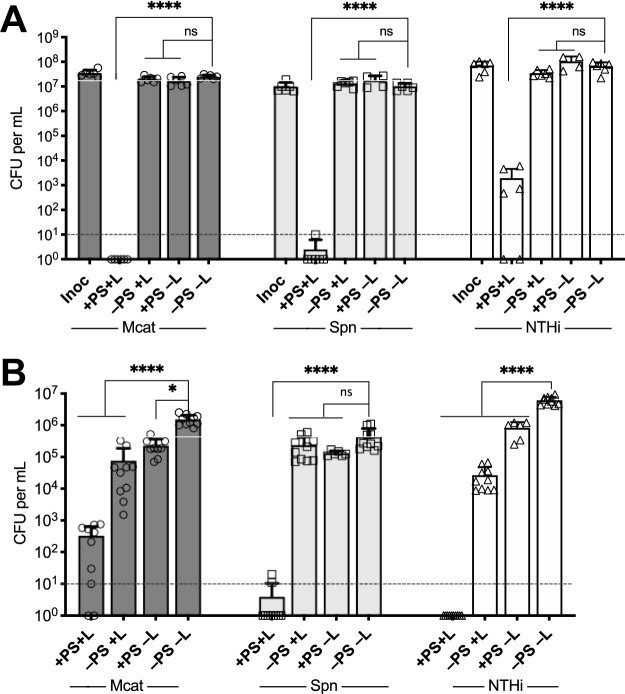
Bactericidal effects of Ce6-aPDT on planktonic cultures (A) and established biofilms (B) of M. catarrhalis 7169 (Mcat), S. pneumoniae EF3030 (Spn), and NTHi 86-026NP (NTJi). Total light dosage was 12 J/cm^2^ (100 mW/cm^2^, 2 min) using 1 μM Ce6 for all planktonic assessments and 90 J/cm^2^ (100 mW/cm^2^, 15 min) using 10 μM Ce6 for biofilm assays. Control sets were exposed to Ce6 but not light (+PS–L), light in the absence of Ce6 (–PS+L), or were not treated (–PS–L). Survival of bacteria, both planktonic and biofilm-associated, was determined by viable plate counts. All bacteria exposed to aPDT treatment (+PS+L) were significantly decreased in viability compared to that of the controls (–PS–L) with a >99.9% kill rate. The dashed lines indicate the limit of detection. The data are presented as the means ± SDs, with individual data points shown. ns, not significant; *, *P = *0.0327; ****, *P* < 0.0001.

We investigated the efficacy of Ce6 in aPDT versus clinical isolates of the three major otopathogens responsible for the majority of acute OM (AOM) cases. The highly effective bactericidal activity of Ce6-aPDT ranged from a 4- to 7-log_10_ decrease in viability (corresponding to a minimum kill rate of 99.99%) against these isolates grown planktonically (Fig. 1A) and as biofilms (Fig. 1B). These data confirmed this approach may be broadly effective as a novel antimicrobial therapeutic for bacterial OM. In particular, antibiofilm efficacy against otopathogenic bacteria is critical, as previous studies have demonstrated that biofilm formation occurs clinically *in vivo* within the middle ears and on the adenoids of pediatric OM patients and that biofilm-associated organisms are often refractory to antibiotic treatments and responsible for disease recurrence (13–16).

### Posttreatment viability of biofilm-detached bacteria.

**FIG 2:**
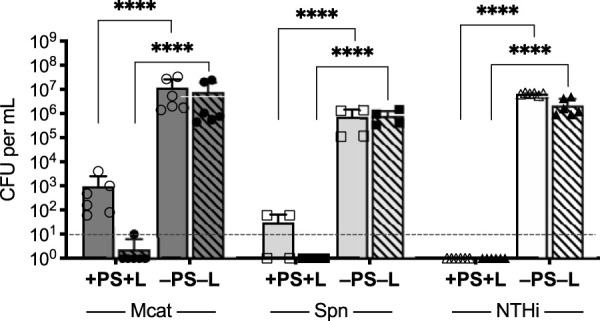
Viability of biofilm-detached bacteria (hatched bars) was assessed by evaluating surviving CFU in the surrounding saline supernatant following aPDT of M. catarrhalis 7169 (Mcat), S. pneumoniae EF3030 (Spn), and NTHi 86-026NP (NTHi) biofilms (solid bars). There were no surviving bacteria (≤10 CFU) detected in the surrounding saline following Ce6-aPDT of the biofilms compared to the untreated control groups (≥10^6^ CFU), indicating that bacteria that detach from the biofilms in response to aPDT are not viable. The dashed line indicates the limit of detection. The data are presented as the means ± SDs, with independent replicates shown. ****, *P* < 0.0001.

**FIG 3:**
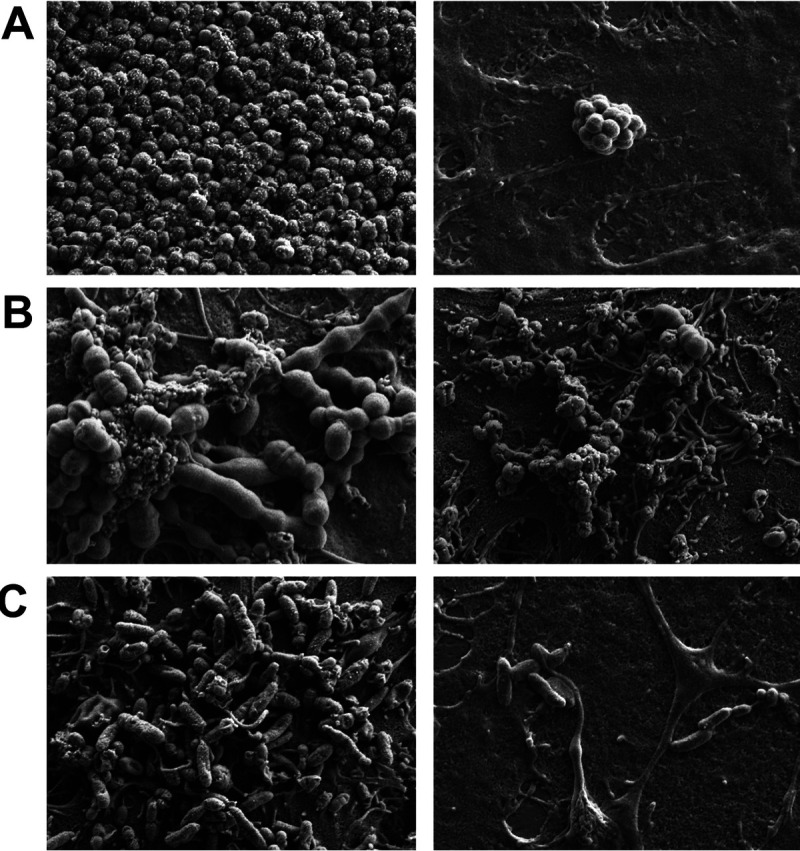
Representative SEM images (×5,000 magnification) of M. catarrhalis 7169 (A), S. pneumoniae EF3030 (B), and NTHi 86-026NP (C) biofilms before (left) and after (right) Ce6-aPDT treatment.

As biofilm-associated bacteria may be released into the environment and colonize new surfaces or cause disseminated disease, we investigated whether the observed decrease in biofilm-associated bacterial CFU following aPDT were due to the loss of cell viability or bacterial detachment from the biofilm and release into the surrounding environment. The viability of post-aPDT biofilm-detached bacteria was assessed by enumerating surviving CFU in the reaction well supernatants. As shown in Fig. 2, the data indicate the antimicrobial activity of Ce6-aPDT is bactericidal for the biofilm-associated bacteria and that there were no surviving detached bacteria above the level of detection, representing a >99.99% kill rate, compared to that in untreated control wells. Figure 3 presents representative scanning electron microscopy (SEM) images of otopathogen biofilms before and after aPDT treatment. Qualitative decreases in attached bacterial biomass for all three strains were visibly detected following Ce6-aPDT treatment.

### Efficacy of a single aPDT treatment versus two sequential aPDT treatments.

**FIG 4:**
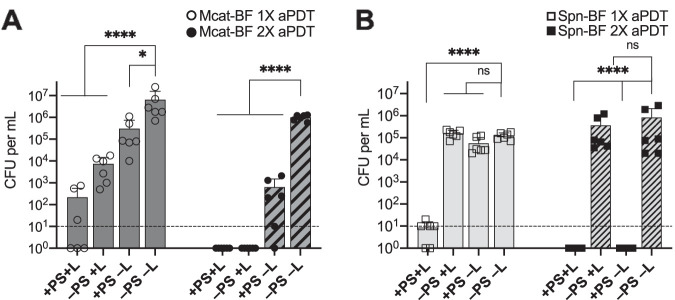
Efficacy of two sequential treatments of Ce6-aPDT was evaluated on biofilms of M. catarrhalis 7169 (A) and S. pneumoniae EF3030 (B). NTHi 86-026NP biofilms were omitted from this analysis because the single aPDT treatment was sterilizing. Biofilms were subjected to one round of Ce6-aPDT (1× aPDT at a total light dosage of 90 J/cm^2^ using 10 μM Ce6; solid bars) or immediately retreated with a second round (2× aPDT) using the exact same treatment parameters (hatched bars). Dual treatment completely eradicated all viable biofilm-associated bacteria (≤10 CFU) compared to untreated controls (≥10^5^ CFU) in both organisms. The dashed lines indicate the limit of detection. The data are presented as the means ± SDs, with independent replicates shown. ns, not significant; *, *P = *0.0281; ****, *P* < 0.0001.

The observed bactericidal activity of Ce6-aPDT versus biofilm-associated otopathogenic bacteria was significant, with a >4- to 6-log_10_ decrease in bacterial viability. Moreover, the treatment was completely sterilizing against NTHi biofilms. However, in the cases of M. catarrhalis and S. pneumoniae, although the treatment resulted in statistically significant reductions in bacterial burden (99.9% clearance rate), viable bacteria remained biofilm associated following treatment. Even low numbers of surviving bacteria have the potential to perpetuate chronic or recurrent disease. Therefore, based on these results, we investigated the efficacy of performing a second cycle of Ce6-aPDT immediately following the initial treatment on the survival of biofilm-associated M. catarrhalis (Fig. 4A) and S. pneumoniae (Fig. 4B). The dual treatment parameters completely eradicated the remaining viable organisms and resulted in a sterilizing effect, with no viable organisms detected via CFU enumeration for both M. catarrhalis and S. pneumoniae.

### Bacterial regrowth following aPDT of established biofilms.

**FIG 5:**
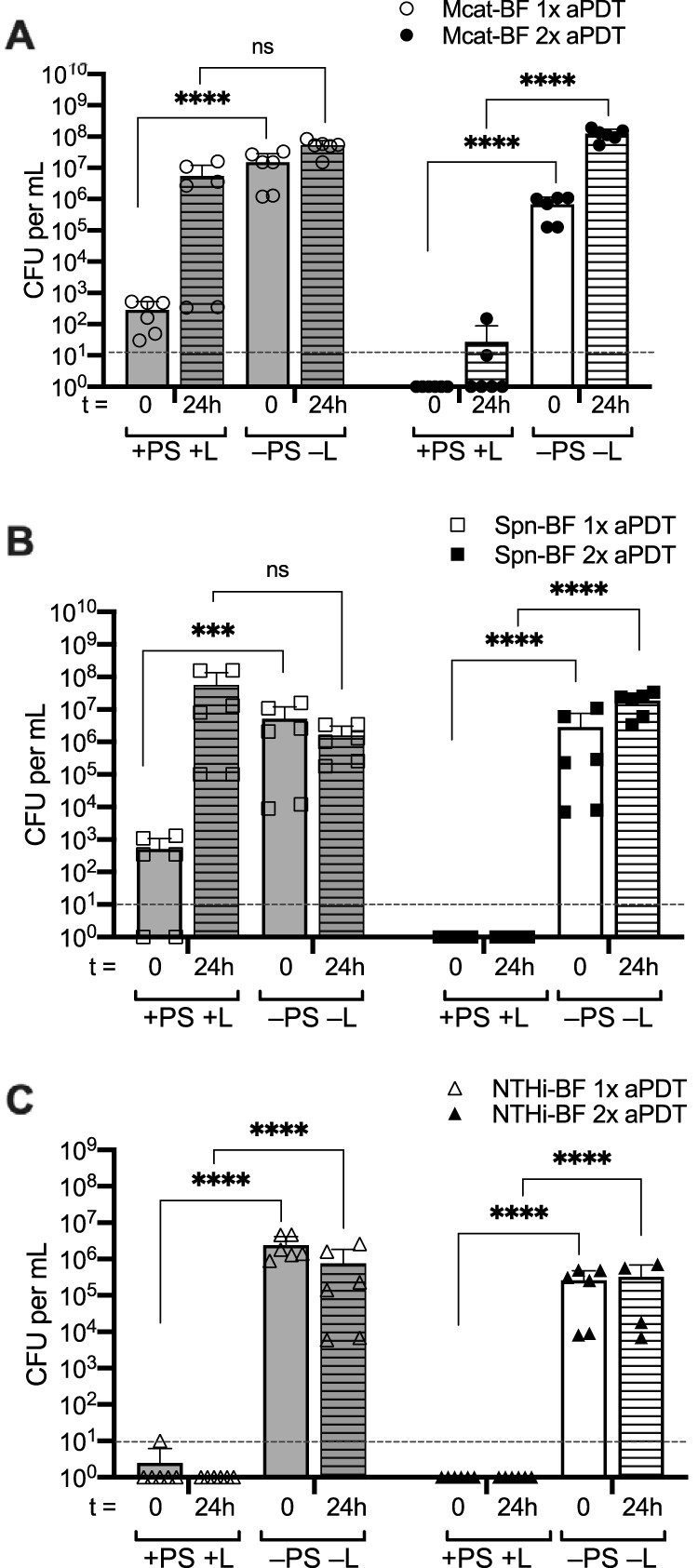
Ce6-aPDT is effective at preventing bacterial regrowth and biofilm reestablishment 24 h following dual treatment. Biofilms of M. catarrhalis 7169 (A), S. pneumoniae EF3030 (B), and NTHi 86-026NP (C) were exposed to a single (gray bars, open symbols) or dual (white bars, closed symbols) Ce6-aPDT treatments (total light dosage of 90 J/cm^2^ using 10 μM Ce6). The surviving biofilm-associated bacteria were enumerated to assess viability immediately following treatment (*t* = 0, solid bars) or 24 h posttreatment (t = 24 h, lined bars). The dashed lines indicate the limit of detection. The data are presented as the means ± SDs, with independent replicates shown. ns, not significant; ***, *P = *0.0008; ****, *P* < 0.0001.

To further extend this finding, we evaluated the regrowth and biofilm formation potential of the bacteria subjected to single or dual treatment modalities. As shown in Fig. 5, although sterilization of the NTHi wells was maintained for 24 h following a single Ce6-aPDT treatment, significant regrowth and biofilm formation were detected for both M. catarrhalis and S. pneumoniae 24 h posttreatment. Significantly, the dual treatment regimen was highly effective in maintaining biologically relevant decreases in bacterial viability for both of these otopathogens, strongly supporting the potential of dual-treatment Ce6-aPDT to effectively decrease or eliminate bacterial survival in established otopathogen biofilms associated with recurrent OM disease.

## DISCUSSION

OM is initiated by the introduction of otopathogens residing as part of the nasopharyngeal microbiota into the normally sterile middle ear cavity. Subsequent inflammatory responses and the establishment of bacterial biofilms within this confined anatomical space not only initiate the acute disease process but also set the stage for disease recurrence. As with all biofilm-based diseases, infection resolution is compromised by the inherent resistance of the biofilm-associated bacteria to both conventional antibiotic therapy and immune system responses, thus enabling the persistence of chronic or recurrent OM infections.

In the present study, we evaluated the efficacy of aPDT using the PS Ce6 and blue light against planktonic cultures and biofilms formed by the three most clinically prominent otopathogens. We observed that a single aPDT treatment significantly decreased bacterial viability by >99.9% for all three bacterial strains. It is well established that biofilm-associated bacteria are typically less sensitive to aPDT than planktonic cells (17, 18). Thus, it was not unexpected that PS concentration and irradiation times had to be increased to achieve similar photoinactivation rates within the biofilms. In addition, higher PS concentrations (up to 100 μM) did not yield an increase in treatment efficacy (data not shown), possibly due to drug aggregation or optical shielding of light penetration as described previously (9). However, it was an initially unexpected finding that NTHi exhibited increased sensitivity to Ce6-aPDT when grown as a biofilm versus as planktonic cultures. Previous studies have documented a difference in protocol efficacy on some microorganisms depending on growth culture conditions and have postulated that microbial suspensions may diffract the penetration potential of the light source, cellular aggregates may interfere with PS binding or access to the cellular membrane, and/or the structure and composition of the biofilm matrix may enhance PS affinity (19–21), likely explaining the observed results with the Ce6-aPDT parameters used in this study on NTHi.

It has been stated that the ideal aPDT therapeutic approach is to use a PS at the lowest effective concentration such that the antimicrobial effect is light potentiated (22, 23). The marked microbial reduction rate of the antibiofilm therapeutic approach we report herein can be characterized as having a significant bactericidal effect, which is defined as a 3-log_10_ decrease in CFU per ml (corresponding to a 99.9% kill rate) (24–26). Furthermore, our results indicate the decrease in biofilm-associated bacteria was the result of bacterial killing by the Ce6-aPDT protocol, as no viable detached bacteria were detected in the surrounding medium. However, we also demonstrated that the single treatment parameters were insufficient to prevent reestablishment of the biofilm 24 h following the delivery of treatment. This finding is similar to the clinical recurrence of OM following completion of a standard antimicrobial regimen as a result of a small number of surviving biofilm-associated organisms.

Thus, to extend our study, biofilms from all three otopathogens were subjected to a second cycle of Ce6-aPDT. The efficacy of this protocol in decreasing bacterial load and persistence in biofilms was confirmed by evaluating regrowth 24 h following treatment delivery. The sustained reduction of viable bacteria following the dual-treatment protocol is highly significant and encouraging. Our results show that dual treatment of otopathogen biofilms with Ce6-aPDT effectively inhibits bacterial regrowth and the reestablishment of intact biofilms and eradicates free-living nonattached bacteria that would have the ability to disseminate or reestablish infection. Our finding that dual Ce6-aPDT treatment cycles effectively prevent bacterial regrowth and biofilm formation is, to our knowledge, the first report of using sequential application of aPDT to attain sustained clinically relevant reductions or complete eradication of biofilm-associated bacteria. Moreover, this highly efficient photoinactivation approach achieved a disinfecting effect, which is defined as a >5-log_10_ decrease in microbial inactivation (corresponding to a >99.999% viability reduction) according to infection control guidelines (27, 28).

Although the general decreased sensitivity of Gram-negative organisms, compared to that of Gram-positive bacteria, to aPDT is well documented (reviewed in references 28–30), our data do not reflect this using the Ce6-aPDT protocol we established. The comparative experiments evaluating the sensitivity of Gram-negative M. catarrhalis and NTHi and Gram-positive S. pneumoniae, using low concentrations of PS and constant light intensity and irradiation periods, indicate highly successful inactivation of both planktonic and biofilm-associated organisms irrespective of Gram classification. However, although we obtained superior and sustained eradication following the dual-treatment protocol, dark toxicity of the PS toward S. pneumoniae became evident, consistent with previous reports describing a bactericidal effect of PSs alone, in the absence of illumination, toward Gram-positive organisms due to a more rapid intracellular drug accumulation (29, 31–33). Likewise, the observed antimicrobial effect of light exposure alone has been demonstrated in multiple bacterial species, with Gram-negative bacteria often showing increased sensitivity to photoinactivation, which has been attributed to the presence of endogenously synthesized cellular porphyrins that act as native PSs and, upon light activation, cause lethal ROS production (21, 32, 34, 35). Thus, our data suggest the application of aPDT is an effective antimicrobial strategy that both interferes with maintenance of bacterial viability and disorganizes and compromises the integrity of the biofilm structure. This photodynamic degradation of the biofilm matrix increases the susceptibility of surviving CFU to the second round of aPDT treatment, which had a sustained disinfecting effect.

Despite the encouraging *in vitro* results of this study, clearly more work must be performed to determine the overall feasibility and efficacy of this approach. This study focused on evaluating and maximizing the bactericidal activity of aPDT versus monomicrobial biofilms, and although monomicrobial otopathogen AOM infections have been reported, future work should involve multispecies otopathogen biofilms for optimal middle ear (ME) clinical disease relevancy. However, it is also important to note that our data have established a foundation that will provide the starting parameters for these more complex polymicrobial biofilm studies. Second, whereas quantitative log-reduction calculations were performed following Ce6-aPDT to assess bacterial viability, the effect of these treatment parameters on human middle ear cells remains to be evaluated. Moreover, the antimicrobial effect of Ce6-aPDT could be potentiated by the addition of relevant antimicrobials and should also be evaluated. A synergistic combination could allow for further reductions in light-emitting diode (LED) exposure time and Ce6 incubation time and concentration, thus reducing any potential phototoxicity to host cells. As the bactericidal activity of aPDT is based on the direct injury of target cells by the production of reactive oxygen species, the potential for the development of resistance is highly unlikely. However, the potential effect of limited oxygen in the ME cavity during OM on aPDT efficacy must also be considered.

Overcoming the physical barrier of the tympanic membrane requires additional attention for optimal treatment efficacy and feasibility. The Ce6 must be delivered to the ME, but this can be readily achieved via tympanostomy tubes, which are often utilized in children with recurrent OM, and/or tympanic membrane-permeating topical carrier agents. Illumination of the ME through the tympanic membrane must also be considered. It is important to note that using the current aPDT parameters outlined in this report, the distance the light travels and total surface area and volume illuminated are significantly larger than the area of the pediatric ear canal and ME space. Given the above-mentioned facts, future studies designed to address the efficacy of our Ce6-aPDT treatment delivered through a barrier into a fluid-filled cavity containing the target biofilms to model the ME *in vitro* are warranted. These should include smaller areas of treatment, smaller volumes of fluid, and the use of fluids more physiologically relevant to ME effusions. Importantly, the confined nature of the ME cavity will likely minimize dilution and diffusion effects while enhancing the interaction between the PS and the target bacteria. Additional *in vitro* experiments involving multispecies biofilms under a variety of clinically relevant conditions followed by *in vivo* research are necessary to fully investigate the potential for future clinical implementation of Ce6-aPDT against OM.

In conclusion, the outcomes of this study indicate a significant bactericidal efficacy of Ce6-aPDT against the three major pediatric otopathogens growing planktonically and in established biofilms. Importantly, the observed maintenance of clearance and inhibition of bacterial regrowth following treatment is a significant and novel aspect of our study, as most aPDT studies do not evaluate this critical outcome. Our data provide the foundation for further studies designed to determine if this innovative therapeutic strategy can be adapted for use against OM infections. Additional studies are warranted to evaluate the potential of Ce6-aPDT to completely disrupt established polymicrobial biofilms, either alone or as an adjuvant strategy to increase the efficacy of standard antibiotic treatments, with the goal of decreasing retractable and recurrent OM disease.

## MATERIALS AND METHODS

### Culture conditions.

The clinical pediatric otopathogens used in this study were previously described and extensively studied. M. catarrhalis 7169 and S. pneumoniae EF3030 (serotype 19F) are otitis media isolates originally obtained via tympanocentesis (36, 37); NTHi 86-026NP was isolated from the nasopharynx of a chronic OM patient (38). Unless otherwise delineated, bacteria were routinely cultured aerobically in broth at 37°C or on agar plates at 37°C in 5% CO_2._ M. catarrhalis was cultured using Mueller-Hinton broth or agar plates, NTHi was grown in brain heart infusion broth supplemented with 2 mg per ml heme and 2 mg per ml NAD (sBHI) or on chocolate agar plates, and S. pneumoniae was incubated statically in a chemically defined bacterial growth medium (CDM) as described (39) or on blood agar plates. NCI-H292 bronchial epithelial cells (ATCC CCL-1848) were grown and fixed using 4% paraformaldehyde on 24-well plate bottoms as described previously (39, 40).

### Biofilm formation on fixed epithelial substratum.

It was previously demonstrated that although pneumococci rapidly kill epithelial cells in the absence of a host response, pneumococcal biofilms formed on prefixed NCI-H292 mucoepidermoid bronchial carcinoma cells share morphology and general architecture with biofilms formed *in vivo* (41). Thus, this *in vitro* model system, which utilizes a fixed epithelial substratum to simulate the epithelial cell-bacterial cell interactions that occur during biofilm formation in the respiratory tract, was used in this study for evaluation of Ce6-aPDT efficacy against otopathogen biofilms. Static bacterial biofilms were grown as previously described (40, 41). In brief, 1 × 10^7^ CFU were resuspended in CDM and seeded on a fixed H292 cell substratum and incubated at 34°C (human nasopharyngeal temperature) for 24 h (M. catarrhalis and NTHi) or 48 h (S. pneumoniae), with changes of culture medium at 12-h intervals, to allow for stable and mature biofilm development. A minimum of three independent biofilm assessments with at least two replicates each were performed per bacterial strain.

### Photosensitizer.

High-purity (>98%) lyophilized chlorin e6 trisodium salt (Ce6) (Fotolon; Apocare Pharma GmbH, Bielefeld, Germany) was handled in the dark to restrict ambient light exposure. A stock solution of Ce6 (6 mg per ml) was prepared in double-deionized sterile water (ddH_2_0) and passed through a 0.2-μm filter (Pall Corporation, Ann Arbor, MI). Single-use aliquots were stored at −20°C. Working concentrations (1 μM to 100 μM) were prepared in sterile physiologic saline (SPS; 0.9% NaCl, pH 7.2) immediately prior to the start of each experiment.

### Light source parameters.

Bacteria were illuminated with a continuous-wave 405 ± 10-nm light-emitting diode delivered from a Liquid Light Guide (LLG-5) Coupled Ultra High-Power UV LED (UHP-F5-405; Prizmatix, Giv'at Shmuel, Israel) for all experiments. Power output of the LED was measured prior to and following exposures using an Optical Power and Energy Meter (PM160; Thorlabs, Germany). The distance between the LED aperture and the target was set at 21.5 cm to allow for the simultaneous illumination of 4 wells. The average light intensity delivered to each reaction well was 100 mW/cm^2^. For the optimal efficacy protocols determined by the preliminary experiments and presented in this report, planktonic cultures were exposed to a total light dose of 12 J/cm^2^ (100 mW/cm^2^, 2 min), and biofilm experiments had total light exposure of 90 J/cm^2^ (100 mW/cm^2^, 15 min). The dosage applied to the sample was calculated as *E* = *Pt*, where *E* is the dose in J/cm^2^, *P* is irradiance in W/cm^2^, and *t* is time in seconds (42).

### aPDT procedures.

Bacterial cultures were resuspended in SPS to an optical density at 600 nm of 0.1 to generate the inoculum used for planktonic (corresponding to ∼1 × 10^7^ CFU/ml) aPDT assays; 1-ml aliquots were placed into the wells of a 24-well plate. The CFU per milliliter of the starting inoculum for every experiment was determined by dilution plating. Biofilms were established as described above. Supernatants from preformed biofilms were removed, and the biofilms were washed with SPS and then overlaid with 1 ml fresh SPS. Wells were inoculated, in the dark, with freshly diluted Ce6 and incubated as described at 25°C. Prior to irradiation, drug was removed from the wells, and the biofilms were gently washed with SPS and overlaid with 1 ml fresh SPS. Preliminary experiments were conducted to determine the effect of incubation time (2 min to 30 min), drug concentration (1 μM to 100 μM), and irradiation time (30 s to 30 min) on aPDT efficiency; based on these data, we selected the optimal parameters for all subsequent experiments. Control samples were exposed to the light in the absence of Ce6 (−PS+L), incubated with Ce6 without exposure to the light (+PS−L), or incubated in SPS alone (−PS−L; saline control). Following treatment, the surrounding saline within each well was transferred to sterile dilution tubes (to enumerate the biofilm-detached organisms), and the biofilms were washed with 1 ml SPS-0.1% saponin and placed in a sonicating water bath followed by mechanical disruption to remove any remaining biofilm mass. All planktonic, biofilm-detached, and biofilm experimental samples were dilution plated in triplicates to enumerate viable CFU. For dual-treatment experiments, the aPDT protocol described above was performed with the following modifications. Immediately following completion of the initial aPDT treatment, only the surrounding saline was harvested from each well; the biofilms were not disturbed or harvested for surviving CFU enumeration. Instead, replicate wells were inoculated with freshly diluted aliquots of drug within 1 min of the completion of the first LED exposure period and the entire aPDT procedure (incubation with 10 μM Ce6 for 15 min followed by exposure to a total light dose of 90 J/cm^2^ [100 mW/cm^2^, 15 min] before quantitation of surviving bacteria) was repeated for +PS+L samples as well as +PS−L, −PS+L, and −PS−L controls. To assess regrowth following single or dual aPDT treatments, wells were processed as above for time zero (*t* = 0) time points. For 24-h (*t* = 24 h) time points, fresh medium was added to washed wells immediately following Ce6-aPDT treatment, and enumeration of CFU followed 24 h of incubation at 37°C in 5% CO_2_. Three to nine independent replicates were performed on separate days with independently grown cultures for each reaction condition per strain per assay. Bacteria were visualized by scanning electron microscopy (SEM) at the UB Instrumentation Center with a Hitachi SU-70 SEM as described (43).

### Statistical analyses.

Data are presented as means ± the standard deviations (SDs). Statistical significance was determined between each of the treated groups compared to the nontreated control using a one‐way analysis of variance (ANOVA) followed by Dunnett’s multiple-comparison tests on log-transformed data (as bacterial CFU follow log-normal distributions [44–46]) using GraphPad Prism 8 software. An unpaired two-tailed *t* test was used to compare the viability of biofilm-detached versus biofilm-associated cells and culture regrowth assessments. A *P* value of <0.05 was considered significant.
